# Prognostic Relevance and Immune Correlates of DAPK1 Expression and CD4^+^/CD8^+^ T-Cell Infiltration in Oral Squamous Cell Carcinoma

**DOI:** 10.3390/cells15171566

**Published:** 2026-08-28

**Authors:** Hsiao-Chi Lai, Keng-Ming Chang, Ching-Chih Lee

**Affiliations:** 1 Department of Medical Education and Research, Kaohsiung Veterans General Hospital, Kaohsiung 813414, Taiwan; 2 Department of Pharmacy and Master Program, College of Pharmacy and Health Care, Tajen University, Pingtung 907101, Taiwan; 3 Department of Otolaryngology, Head and Neck Surgery, Kaohsiung Veterans General Hospital, Kaohsiung 813414, Taiwan; 4 Department of Otolaryngology, Head and Neck Surgery, Pingtung Veterans General Hospital, Pingtung 900053, Taiwan; 5 School of Medicine, National Defense Medical Center, Taipei 114036, Taiwan; 6 Institute of Hospital and Health Care Administration, National Yang Ming Chao Tung University, Taipei 112304, Taiwan; 7 Department of Early Childhood Care and Education, Cheng Shiu University, Kaohsiung 833301, Taiwan

**Keywords:** oral cancer, death-associated protein kinase 1 (DAPK1), overall survival, immune infiltration, apoptosis, head and neck squamous cell carcinoma

## Abstract

Background: Death-associated protein kinase 1 (DAPK1) is a key regulator of apoptosis and immune responses; however, its prognostic significance in oral cancer remains insufficiently characterized. This study investigated the prognostic relevance of DAPK1 in oral squamous cell carcinoma (OSCC) and examined its associations with immune infiltration and apoptosis-related signaling pathways. Methods: A retrospective translational study design was employed, integrating TCGA-based expression and methylation analyses of 528 head and neck squamous cell carcinoma (HNSCC) tumors, UALCAN epigenetic profiling, GeneMANIA protein–protein interaction mapping, TIMER 2.0 immune correlation analyses in 422 HPV-negative HNSCC patients, and multiplex immunofluorescence validation using a tissue microarray cohort of 82 patients with histologically confirmed OSCC, of whom 75 were eligible for the final analysis at Kaohsiung Veterans General Hospital, Taiwan. Results: In vitro validation using Western blot analysis in FaDu cells showed that epidermal growth factor receptor (EGFR) inhibition with gefitinib induced upregulation of DAPK1 protein expression at 10 μM and increased total caspase-3 expression. Higher DAPK1 signal in whole-field quantification was associated with increased CD4+ and CD8+ T-cell infiltration and enrichment of apoptosis-related pathways. Patients with high DAPK1 expression demonstrated a consistent protective trend for overall survival in a pre-specified fully adjusted primary model (adjusted HR = 0.51, 95% CI: 0.21–1.21, *p* = 0.126), and exhibited significantly improved survival in a secondary parsimonious model (adjusted HR = 0.41, 95% CI: 0.18–0.91, *p* = 0.029). Multiplex immunofluorescence further confirmed stronger DAPK1 and caspase-3 staining, along with denser lymphocytic infiltration within the tumor microenvironment. Conclusions: Collectively, these findings suggest that DAPK1 is associated with apoptosis-related signaling, increased immune-cell infiltration, and favorable clinical outcomes in OSCC, although its independent prognostic value requires validation in larger cohorts.

## 1. Introduction

In Taiwan, oral cancer in men is strongly associated with prolonged exposure to carcinogenic risk factors, particularly tobacco, alcohol, and betel nut consumption [1]. More than half of patients are diagnosed at advanced stages of disease, contributing to substantial therapeutic challenges and poor clinical outcomes [2,3]. Although considerable advances have been achieved in radiotherapy techniques, such as intensity-modulated radiotherapy (IMRT), systemic therapies, including epidermal growth factor receptor (EGFR) inhibitors (cetuximab, gefitinib), and immunotherapy (nivolumab, pembrolizumab) [4,5,6], intrinsic and acquired therapeutic resistance remains a major clinical challenge in Taiwan and globally. Consequently, the overall survival (OS) rate of oral cancer patients has not improved substantially over recent decades [7,8]. These limitations underscore the need to identify reliable prognostic biomarkers and novel therapeutic targets associated with tumor progression and immune regulation in oral cancer.

Death-associated protein kinase 1 (DAPK1), a serine/threonine kinase with established tumor-suppressive properties, plays a critical role in the regulation of apoptosis, autophagy, and tumor microenvironment remodeling across multiple malignancies [9]. Recent immunohistochemical studies have demonstrated reduced DAPK1 expression in oral squamous cell carcinoma (OSCC) and oral leukoplakia compared with normal oral mucosa, supporting its involvement in oral carcinogenesis [10,11]. Additionally, promoter hypermethylation of DAPK1 has been identified as a frequent epigenetic alteration across HNSCC subsites, including oral cavity tumors, and has been associated with altered gene expression and adverse clinical outcomes [12]. Despite these findings, the prognostic significance of DAPK1 in oral cancer remains incompletely understood and somewhat controversial. Although certain The Cancer Genome Atlas (TCGA)-based analyses have linked elevated *DAPK1* mRNA expression with poorer survival outcomes in broader HNSCC cohorts [13], accumulating evidence from methylation and protein expression studies suggests that DAPK1 promoter hypermethylation and reduced protein expression are associated with aggressive tumor characteristics and unfavorable prognosis in hypopharyngeal and oral cancers, consistent with its proposed tumor-suppressive role [12,14]. This discrepancy highlights potential discordance between mRNA transcript levels and functional protein expression in the tumor microenvironment. Furthermore, although multiomics studies implicate DAPK1 in tumor immune dynamics [9], in situ validation of DAPK1 protein expression and its concurrent localization with tumor-infiltrating lymphocytes specifically within OSCC remains scarce.

Accordingly, to resolve the discrepancy between mRNA transcriptomic data and protein-level functionality, the present study investigated the clinical significance of DAPK1 expression in oral cancer by evaluating its gene and protein expression profiles, its associations with apoptosis-related proteins and immune-associated cytokines, and its relationship with OS. To achieve this, we integrated in silico analyses of the HNSCC TCGA dataset with multiplex immunofluorescence staining in a dedicated tissue microarray (TMA) cohort of OSCC patients. Finally, preliminary in vitro experiments were conducted to explore the potential mechanistic link between DAPK1 expression and EGFR inhibition.

## 2. Materials and Methods

### 2.1. Bioinformatics and In Silico Predictive Analysis

Differences in DAPK1 gene expression between tumor tissues and adjacent normal tissues were evaluated using the Gene_DE module of the TIMER 2.0 database. The TCGA HNSCC dataset (TCGA-HNSC), comprising 528 primary tumors with available RNA sequencing data and 44 matched adjacent normal tissue samples, was accessed through the UCSC Xena browser and cBioPortal platforms. Promoter methylation levels of DAPK1 in HNSCC and normal mucosal tissues were analyzed using the UALCAN platform to identify potential epigenetic alterations associated with gene dysregulation. To enhance clinical relevance and minimize confounding effects related to viral oncogenesis, immune infiltration analyses were restricted to a cohort of 422 human papillomavirus (HPV)-negative HNSCC patients obtained from TCGA [15,16].

Besides the above, the relationship between DAPK1 expression and the infiltration levels of six immune subsets (CD8^+^ T cells, CD4^+^ T cells, B cells, neutrophils, macrophages, and dendritic cells) was calculated using Spearman’s rank correlation coefficient. Additionally, a protein interaction network was generated using the GeneMANIA algorithm [17] to evaluate the physical and functional associations between DAPK1 and a selected panel of proteins. The queried proteins included ACIN1, AKT3, APPL1, CASP3, CASP6, CR2, DAPK1, DFFA, DFFB, DGKA, DIABLO, DSG1, FADD, GAS2, IFNA1, IFNAR2, IL15, IL15RA, IL21, IL2RG, STK3, STK26, UNC5A, UNC5B, and UNC5C.

### 2.2. In Vitro Western Blot Validation of Epidermal Growth Factor Receptor (EGFR)–DAPK1 Regulation

To experimentally investigate the relationship between EGFR signaling and DAPK1 expression, FaDu human hypopharyngeal squamous cell carcinoma cells (ATCC, Manassas, VA, USA) were cultured in Dulbecco’s modified Eagle medium supplemented with 10% fetal bovine serum and 1% penicillin–streptomycin at 37 °C in a humidified atmosphere containing 5% CO_2_. Cells were treated with gefitinib (Selleck Chemicals, Houston, TX, USA), an EGFR tyrosine kinase inhibitor, at concentrations of 0, 2, 5, and 10 μM for 24 h.

Total cellular protein was extracted using radioimmunoprecipitation assay (RIPA) buffer supplemented with protease and phosphatase inhibitors. Protein lysates (30 μg per lane) were separated by 10% sodium dodecyl sulfate–polyacrylamide gel electrophoresis (SDS-PAGE), transferred onto polyvinylidene difluoride (PVDF) membranes, and incubated with primary antibodies against DAPK1 (Proteintech Group, Inc., Rosemont, IL, USA, 25136-1-AP, 1:1000), EGFR (Proteintech, 18986-1-AP, 1:1000), phosphorylated ERK1/2 (p-ERK) (Cell Signaling Technology, Inc., Danvers, MA, USA, 9101, 1:1000), total ERK1/2 (Cell Signaling Technology, 4695, 1:1000), total caspase-3 (Cell Signaling Technology, 9662, 1:1000), and GAPDH (Invitrogen, Thermo Fisher Scientific, Waltham, MA, USA, AM4300, 1:1000), which served as the loading control. Horseradish peroxidase-conjugated secondary antibodies were subsequently applied, and protein bands were visualized using enhanced chemiluminescence (ECL). Densitometric quantification was performed using ImageJ software (Version 1.53; National Institutes of Health). All experiments were conducted in quadruplicate. Statistical differences were assessed using one-way ANOVA followed by Dunnett’s post hoc test.

### 2.3. Immunofluorescence Staining and Digital Quantification

TMA slides containing duplicate 2.0-mm core biopsy specimens from 82 patients with histologically confirmed OSCC treated at Kaohsiung Veterans General Hospital, Taiwan, between 2012 and 2015, were subjected to multiplex immunofluorescence staining using a previously validated protocol [18]. Primary antibodies included anti-DAPK1 (Affinity Biosciences, Cincinnati, OH, USA, DF8030, 1:200), anti-caspase-3 (Bioss Inc., Woburn, MA, USA, BS-0081R, 1:500), anti-CD4 (Wuhan ServiceBio Co., Ltd., Wuhan, China, GB11064, 1:400), and anti-CD8 (Leica Biosystems, Newcastle upon Tyne, UK, NCL-L-CD8-4B11, 1:100). Species-specific fluorescent secondary antibodies and DAPI nuclear counterstaining were subsequently applied.

Stained slides were scanned using the Vectra Polaris imaging system (Akoya Biosciences, Inc., Marlborough, MA, USA). For quantitative analysis, five randomly selected high-power fields (200× magnification) per core were evaluated in both intratumoral and peritumoral regions. Mean fluorescence intensity (MFI) values for DAPK1 and caspase-3, along with the percentages of CD4^+^ and CD8^+^ cells, were determined following automated segmentation and thresholding using ImageJ software (Version 1.53; National Institutes of Health). Uniform intensity thresholds and background subtraction parameters were applied across all samples [19]. To convert these continuous measurements into categorical variables for downstream comparison, the median values across the entire cohort (n = 75) were defined as the standardized thresholds to stratify patients into ‘high’ and ‘low’ expression/infiltration groups for DAPK1, caspase-3, CD4, and CD8, respectively. After exclusion of cases with incomplete clinicopathological data, 75 patients were included in the final analysis (Supplementary Figure S1).

### 2.4. Statistical Analysis

All statistical analyses were performed using SPSS statistical software (Version 20.0; IBM Corporation, Armonk, NY, USA). Clinical variables analyzed included age, sex, pathological T (pT), and pathological nodal (pN) classifications, tumor thickness (≤4 mm vs. >4 mm), histological differentiation, surgical margin status, perineural invasion, lymphovascular invasion, extranodal extension, and receipt of adjuvant therapy [20].

Clinical staging and pathological T and N classifications were determined according to the AJCC 7th edition, wherein tumor thickness was evaluated as absolute thickness rather than depth of invasion (DOI). Categorical variables were compared using Pearson’s chi-square test or the Fisher–Freeman–Halton exact test, as appropriate based on expected cell counts. Continuous variables were compared using Student’s t-test. Immunofluorescence staining intensity and positive cell counts for DAPK1, caspase-3, CD4, and CD8 were quantified using ImageJ software. For each patient sample, five randomly selected high-power fields (200× magnification) were analyzed. Mean fluorescence intensity (MFI) of DAPK1 was quantified across whole high-power fields without restriction to specific tissue or cellular compartments. Patients were stratified into high- and low-DAPK1 expression groups using the cohort median MFI as the pre-defined primary threshold for dichotomization.

Overall survival (OS), defined as the time from the date of surgical resection to the date of death from any cause or last clinical contact, served as the primary survival endpoint. Survival analyses were not administratively censored at 60 months; standard right-censoring was applied based on the date of last clinical contact. For graphical presentation, the Kaplan–Meier curve was displayed through 60 months. To rigorously account for baseline imbalances, a pre-specified, fully adjusted forced-entry Cox proportional hazards regression model—simultaneously incorporating DAPK1, age, pN, and pT—was conducted as the primary multivariate analysis. Subsequently, a secondary parsimonious model was generated using a backward stepwise selection approach (inclusion criteria: univariate *p* < 0.1). Variables with insufficient event counts, such as lymphovascular invasion (present in only one patient), were excluded from multivariate modeling to prevent statistical instability. A two-sided *p*-value of <0.05 was considered statistically significant, and the proportional hazards assumption was verified for all covariates (Supplementary Table S1).

To assess the robustness of the categorical findings without relying solely on dichotomization, additional post hoc sensitivity analyses were performed using patient-level continuous marker measurements. Receiver operating characteristic (ROC) curve analysis was used to evaluate discrimination for 5-year survival status and to identify the empirically optimal cut-point using the Youden index. In addition, Cox proportional hazards regression was performed with marker MFI entered as a continuous variable after standardization to a Z-score, with hazard ratios expressed per 1-SD increase. These analyses were considered sensitivity analyses and did not alter the pre-specified TMA-based categorical classification used in the primary clinicopathological and survival analyses.

## 3. Results

### 3.1. DAPK1 Is Differentially Expressed Between Tumor and Adjacent Normal Tissues and Exhibits Promoter Hypermethylation in HNSCC

*DAPK1* gene expression across multiple solid tumors was evaluated using the TIMER 2.0 database by comparing primary tumor tissues with adjacent normal tissues. Consistent with recent multiomics investigations in HNSCC, DAPK1 mRNA expression was generally reduced in primary tumors relative to matched adjacent normal tissues (Supplementary Figure S2). The TCGA-HNSC cohort included 528 primary HNSCC tumors with available RNA sequencing data and 44 matched adjacent normal mucosal samples.

To further investigate potential epigenetic mechanisms underlying DAPK1 dysregulation, promoter methylation analysis was performed using UALCAN. DAPK1 promoter methylation levels were significantly higher in HNSCC tumor tissues (n = 528) than in normal mucosal tissues (n = 50, derived from UALCAN’s predefined normal reference set rather than the 44 matched samples used for mRNA analysis; *p* = 1.62 × 10^−12^; Supplementary Figure S3). These findings are consistent with recent reports identifying DAPK1 promoter hypermethylation as a frequent epigenetic alteration across HNSCC subsites and as a potential contributor to adverse clinical outcomes [12].

### 3.2. DAPK1 Is Associated with Apoptosis Pathways and Immune Cell Infiltration

**Figure 1:**
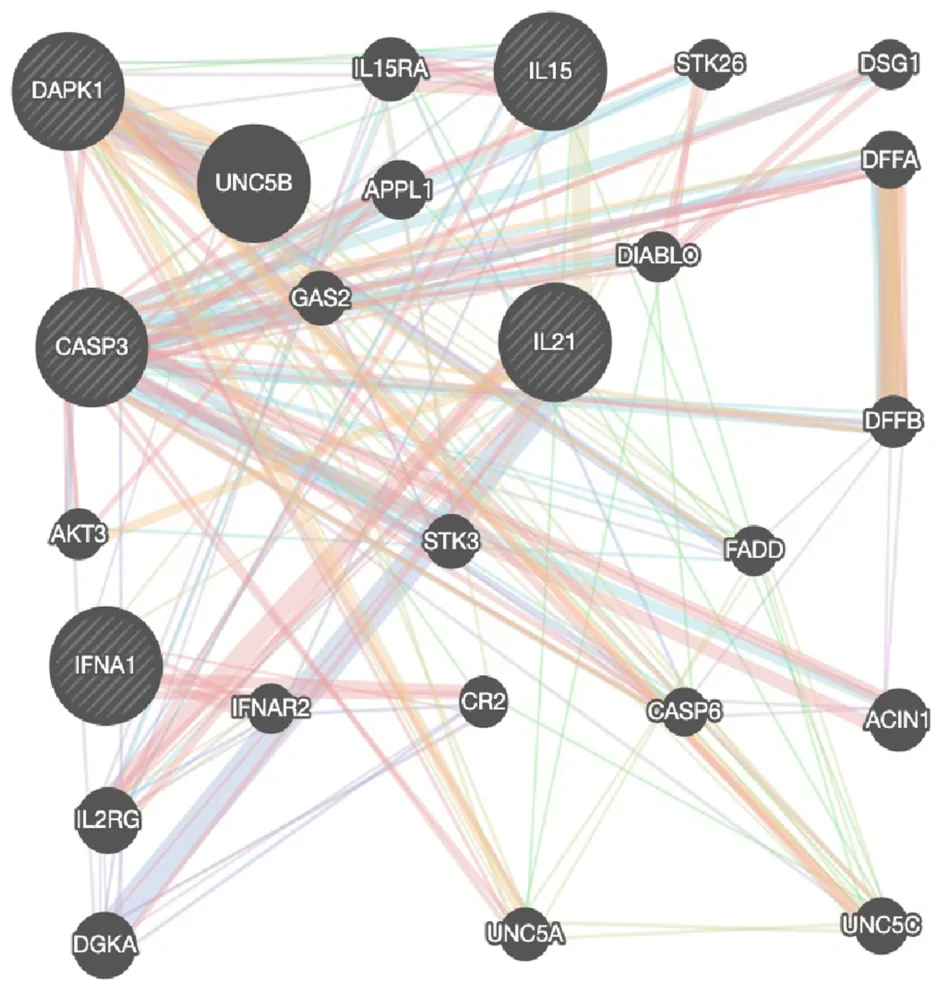
Protein–protein interaction analysis using GeneMANIA revealed the association of DAPK1 with several proteins involved in apoptosis and immune-related cellular processes. Nodes represent the queried proteins, and edges indicate predicted physical or functional interactions.

To explore the functional relevance of DAPK1 in HNSCC, a protein–protein interaction network was generated using GeneMANIA. In silico network analysis suggested potential physical and functional interactions between DAPK1 and several apoptosis-related proteins, including CASP3, as well as immune-associated cytokines such as IL-15, IL-21, and IFNA1 (Figure 1). These bioinformatic predictions provide a theoretical basis to hypothesize that DAPK1 plays a role in the regulation of apoptotic signaling and immune modulation within the tumor microenvironment.

**Figure 2:**
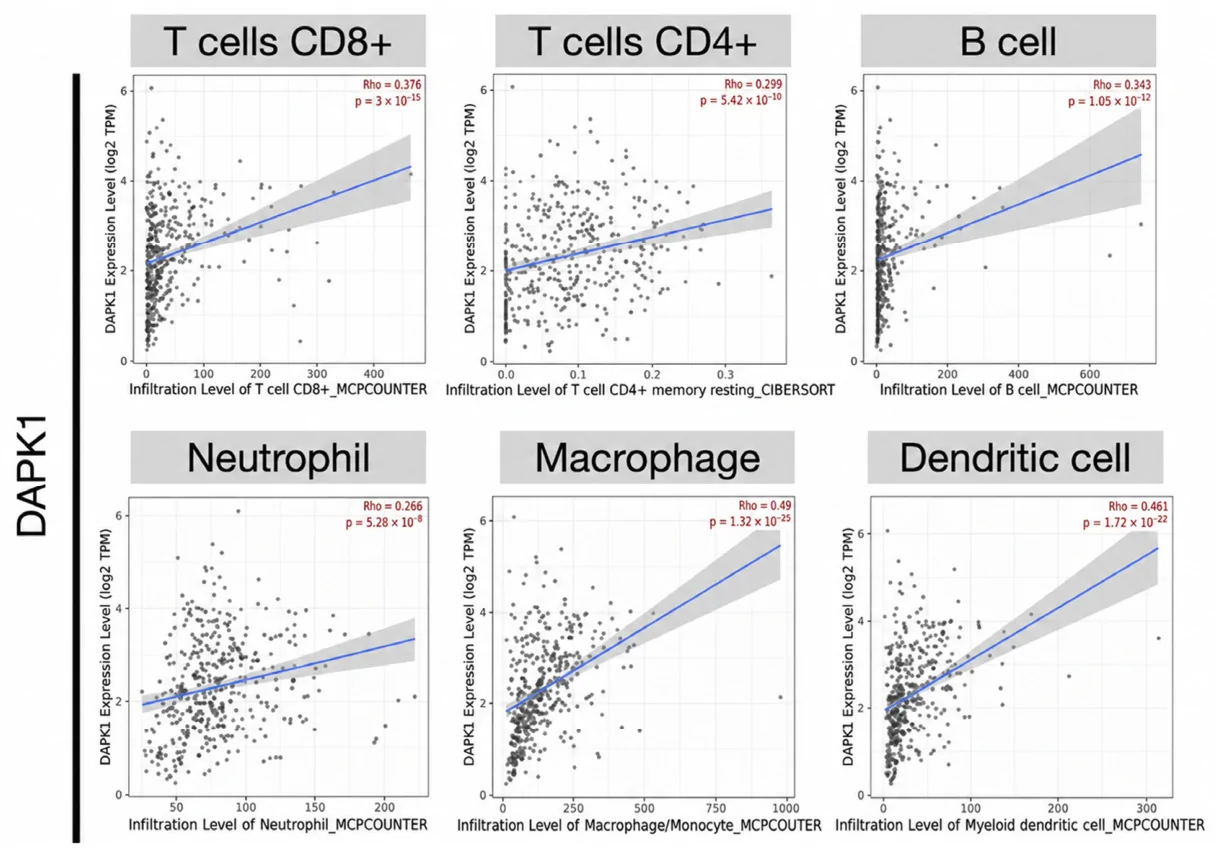
DAPK1 immune infiltration analysis. Scatterplots showing Spearman’s correlations between DAPK1 expression and infiltration of six major immune cell subsets (CD8^+^ T cells, CD4^+^ T cells, B cells, neutrophils, macrophages, and dendritic cells) in 422 HPV-negative head and neck squamous cell carcinoma (HNSCC) patients from the TCGA database. Analysis was performed using TIMER 2.0; positive correlations were observed between DAPK1 expression and infiltration estimates for multiple immune-cell populations (*p* < 0.05).

Subsequently, immune infiltration analysis was conducted in a clinically relevant cohort of 422 HPV-negative HNSCC patients derived from TCGA. TIMER 2.0 analysis demonstrated that DAPK1 expression was positively correlated with infiltration of multiple immune cell populations, including CD4^+^ T cells (CIBERSORT; Rho = 0.299, *p* < 0.001), CD8^+^ T cells (MCPCOUNTER; Rho = 0.376, *p* < 0.001), B cells (Rho = 0.342, *p* < 0.001), neutrophils (Rho = 0.286, *p* < 0.001), macrophages (Rho = 0.490, *p* < 0.001), and dendritic cells (Rho = 0.461, *p* < 0.001) (Figure 2). These findings suggest a potential association between DAPK1 expression and immune-cell infiltration in HNSCC.

### 3.3. DAPK1 Expression Is Associated with Better OS in Patients with Oral Cancer

In the institutional TMA cohort, TMA slides from 82 patients were evaluated. After the exclusion of 7 cases due to incomplete clinicopathological data, 75 patients were retained for analysis. The median MFI thresholds were computed strictly after these exclusions. DAPK1 protein expression was quantified by multiplex immunofluorescence in 75 patients with OSCC who underwent surgical resection between 2012 and 2015 (Supplementary Figure S1). The median follow-up duration for the entire cohort was 60.2 months. During the available follow-up period, a total of 31 deaths from any cause occurred across the cohort. Patients were stratified into high- and low-DAPK1 expression groups according to the median MFI value across all cases (high expression: n = 36; low expression: n = 39). Clinicopathological characteristics are summarized in Table 1.

Categorical variables were compared using Pearson’s chi-square test or Fisher–Freeman–Halton exact test, as appropriate according to expected cell counts. Continuous variables were compared using Student’s t-test. The classification of DAPK1 expression into “high” and “low” groups was determined using the median value as the cutoff point. Abbreviations: SD, standard deviation; DAPK1, death-associated protein kinase 1.

High DAPK1 expression was significantly associated with younger patient age (*p* = 0.033) and lower pN category (*p* = 0.045). To rigorously evaluate potential residual confounding from baseline imbalances, a pre-specified fully adjusted forced-entry Cox model simultaneously incorporating DAPK1, age, pN, and pT was performed as the primary multivariate analysis. In this model, high DAPK1 expression maintained a consistent protective effect direction, although statistical significance was attenuated due to limited event counts across multiple covariates (adjusted HR = 0.51, 95% CI: 0.21–1.21, *p* = 0.126; Table 2). Subsequently, in a secondary parsimonious model using backward stepwise selection, high DAPK1 expression remained an independent predictor of improved OS after adjusting for pT category (adjusted HR = 0.41, 95% CI: 0.18–0.91, *p* = 0.029; Table 2). The proportional hazards assumption was confirmed for all covariates (Supplementary Table S1).

**Figure 3:**
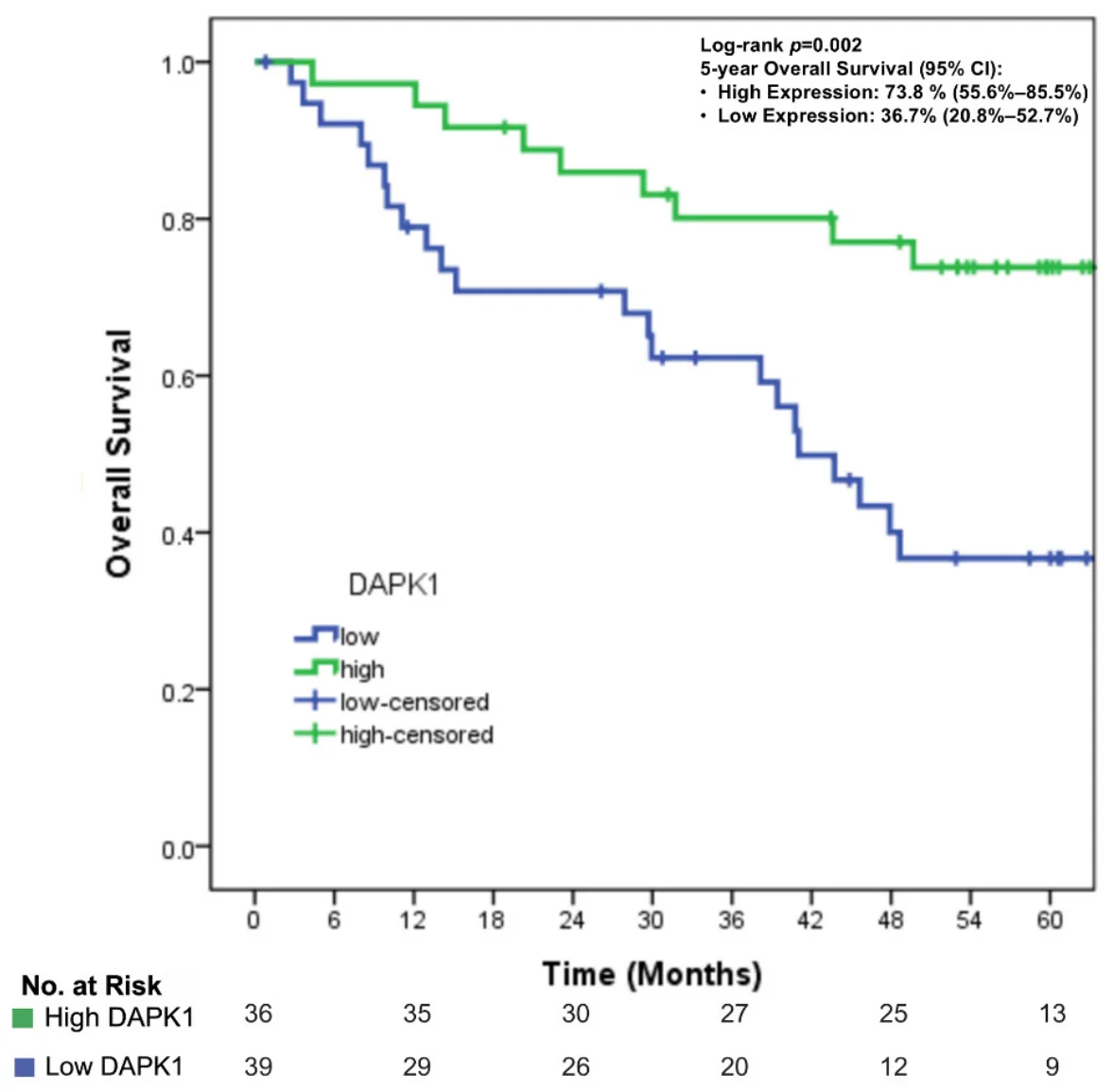
Kaplan–Meier curves for overall survival according to DAPK1 expression in the institutional cohort of 75 patients with OSCC. High and low DAPK1 expression groups correspond to the original TMA-based classification (high, n = 36; low, n = 39). Patients in the high-DAPK1 group demonstrated better overall survival (log-rank *p* = 0.002; 5-year OS: 73.8% [95% CI: 55.6–85.5%] vs. 36.7% [95% CI: 20.8–52.7%]). Numbers at risk are shown below the curves.

Kaplan–Meier survival analysis further demonstrated that patients with high DAPK1 expression, defined as above-median MFI, exhibited significantly better 5-year OS than those with low DAPK1 expression (Figure 3). As an additional assessment of cutoff robustness, a post hoc ROC analysis was performed. Discrimination by DAPK1 MFI alone was modest and did not reach statistical significance (AUC = 0.63, 95% CI: 0.48–0.75). Thus, the ROC analysis should be interpreted as a sensitivity assessment of the pre-defined median-based classification rather than as evidence of strong standalone discrimination (Supplementary Table S2).

### 3.4. EGFR Inhibition Induces DAPK1 Expression and Apoptosis-Related Signaling In Vitro

**Figure 4:**
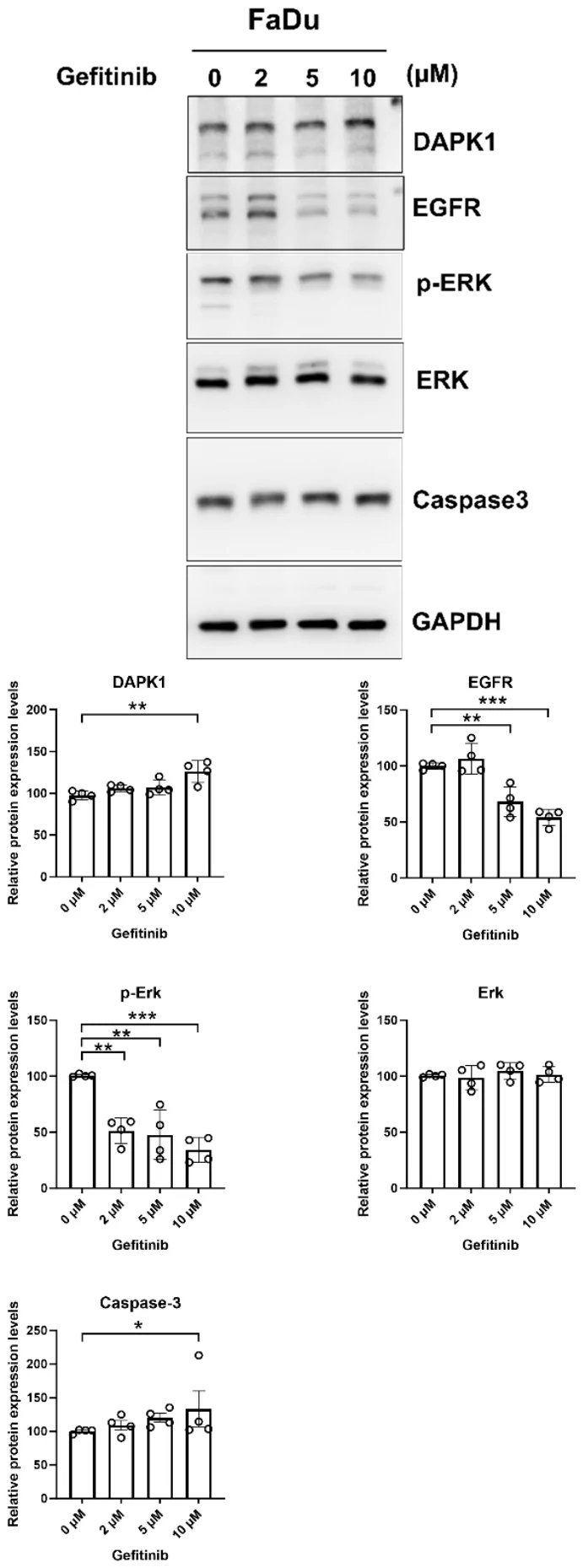
Western blot analysis of FaDu HNSCC cells treated with the indicated concentrations of the EGFR tyrosine kinase inhibitor gefitinib (0, 2, 5, and 10 μM) for 24 h. The cell lysates were immunoblotted to assess the protein expression levels of DAPK1, EGFR, phosphorylated ERK (p-ERK), total ERK, and Caspase 3. GAPDH was used as an internal loading control. Data are presented as mean ± SD from four independent experiments. * indicates *p* < 0.05; ** indicates *p* < 0.01; *** indicates *p* < 0.001 as compared to 0 μM.

To further evaluate the mechanistic relationship between EGFR signaling and DAPK1 expression, in vitro Western blot analysis was performed using FaDu hypopharyngeal squamous cell carcinoma cells treated with the EGFR tyrosine kinase inhibitor gefitinib. Gefitinib treatment (0, 2, 5, and 10 μM for 24 h) resulted in a modest but statistically significant increase in DAPK1 protein expression at 10μM, accompanied by reduced phosphorylated ERK (p-ERK) levels and increased total caspase-3 expression (Figure 4). These findings provide preliminary evidence that EGFR pathway inhibition is associated with increased DAPK1 expression and changes in apoptosis-related signaling.

### 3.5. Expression of DAPK1 Correlates with Markers of Apoptosis and Infiltration of Immune Cells in Oral Cancer

**Figure 5:**
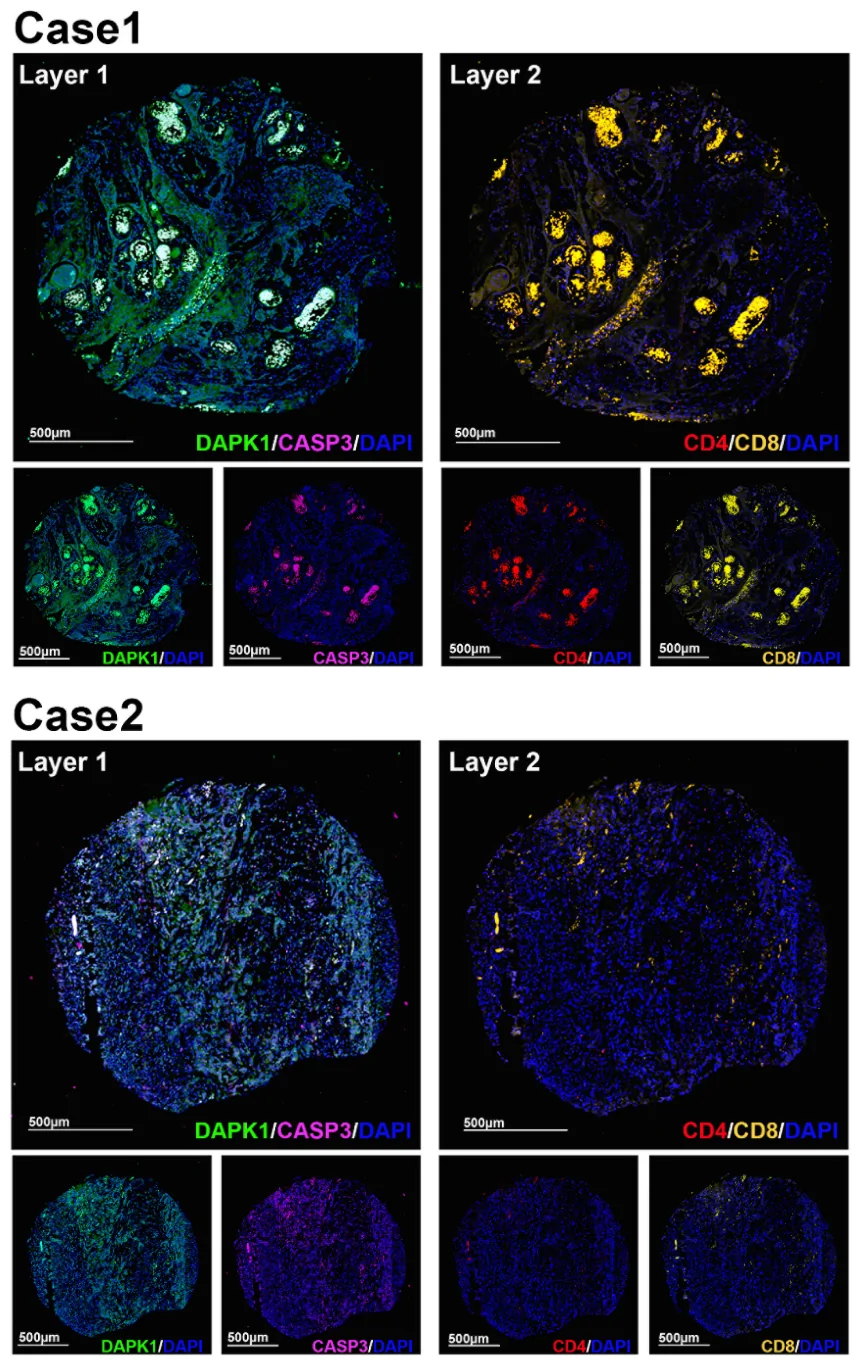
Multiplex immunofluorescent staining demonstrating the relationship between DAPK1 and markers of apoptosis and immune infiltration in the tumor microenvironment. Representative images from two cases: Case 1 (right lower gingival squamous cell carcinoma, pT4aN2bM0, Stage IVA, moderately differentiated) with high DAPK1 expression and Case 2 (right tongue squamous cell carcinoma, pT2N1M0, Stage III, moderately differentiated) with low DAPK1 expression. Layer 1: DAPK1 (green fluorescence), caspase-3 (CASP3, pink fluorescence), and nuclear DAPI counterstain (blue); the white frame shows a magnified inset. Layer 2: CD4 (red fluorescence), CD8 (yellow fluorescence), and nuclear DAPI. The exemplar images were selected for optimal fluorescence clarity to demonstrate concurrent marker expression, rather than strictly representing the cohort’s prevailing pN associations.

Multiplex immunofluorescence staining was subsequently performed on the same TMA cohort to evaluate concurrent expression patterns between DAPK1 expression, apoptotic activity, and immune cell infiltration within the tumor microenvironment. Representative images from a high-DAPK1 expression case (right lower gingival SCC, pT4aN2bM0) and a low-DAPK1 expression case (right tongue SCC, pT2N1M0) are presented in Figure 5.

Layer 1 demonstrated colocalization of green fluorescence (DAPK1) and pink fluorescence (caspase-3) within tumor cells, whereas Layer 2 revealed markedly increased red (CD4^+^) and yellow (CD8^+^) fluorescence signals within both intratumoral and peritumoral stromal regions in the high-DAPK1 expression case. Quantitative analysis across all 75 patients confirmed that high DAPK1 expression was significantly associated with a higher proportion of patients exhibiting high CD4^+^ T cell infiltration (80.6% vs. 41.0%, *p* = 0.001), increased CD8^+^ T cell infiltration (80.6% vs. 17.9%, *p* < 0.001), and elevated caspase-3 expression (86.1% vs. 25.6%, *p* < 0.001; Table 3).

## 4. Discussion

The long-term carcinogenic effects of tobacco, alcohol, and betel nut exposure on DAPK1 expression in the Taiwanese population have not been comprehensively investigated. In the present study, elevated DAPK1 expression in oral cancer was significantly associated with improved OS. Additionally, the findings suggest that DAPK1 may exert antitumor effects through enhancement of apoptotic signaling via regulation of the caspase cascade, as well as through modulation of antitumor immune responses within the tumor microenvironment.

Recent immunohistochemical and methylation-based investigations have further reinforced the tumor-suppressive role of DAPK1 in OSCC and oral potentially malignant disorders. DAPK1 promoter hypermethylation has frequently been identified in OSCC and has been associated with reduced gene expression, advanced disease stage, and unfavorable clinical outcomes [10,12]. These epigenetic alterations are consistent with the present UALCAN methylation analysis (*p* = 1.62 × 10^−12^) and TIMER 2.0 findings and align with broader multiomics investigations demonstrating that DAPK1 methylation-driven gene signatures function as independent prognostic indicators in OSCC [21,22]. Concurrently, elevated DAPK1 protein expression, quantified by multiplex immunofluorescence in the institutional cohort, demonstrated strong associations with increased CD4^+^ and CD8^+^ T cell infiltration and increased total caspase-3 expression. These findings provide an in situ evaluation of the immune-modulatory role of DAPK1 specifically within oral cavity tumors and help address prior inconsistencies arising from TCGA-based HNSCC mRNA analyses [11,23]. Notably, a discrepancy exists between transcriptomic and proteomic findings regarding DAPK1 prognostic value. While some TCGA-based mRNA analyses in broader HNSCC cohorts suggested an adverse prognostic association for high DAPK1 mRNA, the TMA protein-level quantification demonstrates that elevated DAPK1 protein expression was associated with improved 5-year overall survival in OSCC. This mRNA-versus-protein discordance can be attributed to complex post-translational modifications—such as ubiquitin-mediated degradation and phosphorylation—which tightly regulate active DAPK1 protein levels independently of transcription. Therefore, protein-level quantification via multiplex immunofluorescence provides a more biologically accurate indicator of functional DAPK1 within the tumor microenvironment than transcriptomic abundance alone.

Previous investigations have demonstrated that DAPK1 suppresses tumor cell proliferation and metastatic potential through induction of apoptosis across multiple malignancies, including colorectal, breast, and gastric cancers [21,24,25,26,27,28,29]. More recently, Song et al. reported that DAPK1 overexpression induced apoptosis in sunitinib-resistant clear cell renal cell carcinoma through the activation of an ATF6-dependent endoplasmic reticulum (ER) stress pathway [29]. Moreover, Wang et al. [30] demonstrated that exosomal miR-191-5p contributed to radiation resistance in esophageal squamous cell carcinoma through downregulation of DAPK1 via the MAPK–JNK signaling pathway. In the present study, DAPK1 expression demonstrated significant associations with patient age and pN category, supporting its potential prognostic relevance in oral cancer. Importantly, multivariate analysis confirmed that elevated DAPK1 expression was associated with improved 5-year overall survival (adjusted HR = 0.41; 95% CI: 0.18–0.91), with patients exhibiting high DAPK1 expression demonstrating significantly better survival outcomes than those with low expression. Collectively, these findings suggest that DAPK1 may contribute to oral cancer initiation and progression.

To further explore the potential relationship between EGFR signaling and DAPK1, in vitro Western blot analyses were performed in FaDu cells following gefitinib treatment. A modest (~10–20%) significant increase in DAPK1 protein expression was observed following EGFR inhibition at 10μM, accompanied by reduced phosphorylated ERK expression and increased caspase-3 (Figure 4). These findings provide preliminary evidence of an association between EGFR pathway inhibition, DAPK1 expression, and apoptosis-related signaling in HNSCC. Furthermore, the results extend recent clinical observations demonstrating that DAPK1 promoter methylation status may predict sensitivity to anti-EGFR therapies in HNSCC cell lines [14].

DAPK1 is a calcium/calmodulin-regulated serine/threonine kinase that has been implicated in both early tumorigenesis and advanced stages of cancer progression [31]. Previous investigations demonstrated that DAPK1 knockout fibroblasts exhibited reduced expression of ER stress-related proteins, diminished caspase activation, and lower levels of autophagy in response to ER stress [32]. These findings indicate that DAPK1 functions as an important regulator of ER stress, autophagy, and caspase-mediated apoptosis. Consistent with these observations, the present data suggest that DAPK1 may represent a candidate prognostic biomarker in oral cancer through activation of pro-apoptotic signaling pathways and enhancement of tumor cell sensitivity to apoptosis (Table 3).

Moreover, Arantes et al. [14] established HNSCC cell lines that were sensitive or resistant to allitinib. The methylation status of *DAPK1* accurately predicted the treatment resistance in these cell lines (AUC = 0.852), suggesting that DAPK1 may serve as a potential predictive biomarker for response to anti-EGFR therapy in HNSCC [14]. In parallel, recent multiomic investigations have shown that DAPK1 expression is tightly regulated by epigenetic, transcriptional, posttranscriptional, and posttranslational processes that contribute to metastasis and tumor invasiveness [31]. Consistent with these findings, the present UALCAN analysis demonstrated elevated *DAPK1* promoter methylation in HNSCC relative to normal tissues (Supplementary Figure S3), further supporting the clinical relevance of DAPK1-associated epigenetic dysregulation in HNSCC progression.

Emerging evidence has further implicated DAPK1 in the regulation of stromal and immune cell dynamics within the tumor microenvironment. Interestingly, while our multiplex immunofluorescence demonstrated the concurrent expression of DAPK1 with CD4+/CD8+ T cells, our TIMER 2.0 analysis revealed that DAPK1 also correlates strongly with macrophages and dendritic cells. Given that DAPK1-positive macrophages have been previously reported to promote an immunosuppressive microenvironment in colorectal cancer [33], our TIMER 2.0 findings indicate that the observed pattern is at least as consistent with generalized immune-cell infiltration as it is with a T-cell-specific mechanism. Consequently, the distinct and potentially opposing roles of DAPK1 across different immune subsets in OSCC warrant further compartment-specific functional investigations. Cancer stem cells are recognized as major drivers of tumor aggressiveness and metastatic progression through regulation of EMT-associated pathways [34]. The tumor microenvironment comprises tumor cells, cancer-associated fibroblasts, and diverse immune cell populations, including tumor-infiltrating lymphocytes and tumor-associated macrophages [35]. Alterations within this microenvironment can substantially influence tumor behavior and therapeutic responsiveness.

Among immune-related mediators, IL-15 has emerged as a key cytokine capable of enhancing CD8^+^ T cell-mediated antitumor immunity [36]. Recent investigations demonstrated that DAPK1 functions as an essential downstream effector of IL-15 signaling through regulation of the homing receptors CD62L and CCR7 on CD8^+^ T cells, thereby modulating T cell migration and tissue trafficking [37]. Furthermore, in vivo studies revealed that DAPK1 deficiency significantly impaired IL-15-driven antitumor responses, supporting a nonredundant role for DAPK1 in linking cytokine signaling to effector T cell activity [37]. In the present study, elevated DAPK1 expression was strongly associated with increased CD4^+^ and CD8^+^ immune cell infiltration in oral cancer tissues. Multiplex immunofluorescence analysis further demonstrated concurrent expression of DAPK1 with CD4^+^ and CD8^+^ T cells within the tumor microenvironment, findings that were quantitatively corroborated by TIMER 2.0 analyses in 422 HPV-negative HNSCC cases. Collectively, these observations raise the possibility that IL-15-associated signaling may contribute to the relationship between DAPK1 expression and T-cell infiltration; however, this hypothesis was not directly tested in the present study. Nevertheless, additional mechanistic investigations remain necessary to clarify the role of DAPK1 in IL-15-mediated immune infiltration and T cell activation, including studies employing DAPK1-manipulated cellular models and patient-derived specimens.

Taken together, the available evidence indicates that DAPK1 plays a critical role in oral cancer tumorigenesis through enhancement of apoptotic sensitivity and modulation of the tumor immune microenvironment. The present findings demonstrate that elevated DAPK1 expression is associated with improved OS, increased immune cell infiltration, and enhanced apoptotic signaling in oral cancer. These observations further suggest that reduced DAPK1 expression may contribute to activation of pathways associated with metastasis, therapeutic resistance, tumor invasiveness, and ER phagosome-related signaling. In multivariate analysis, tumors characterized by low DAPK1 expression and advanced T-stage disease exhibited the poorest 5-year survival outcomes. Although several molecular mechanisms have been proposed to explain the association between reduced DAPK1 expression and adverse clinical outcomes [29,38], further experimental validation remains necessary to clarify the molecular functions of DAPK1 in oral cancer progression.

Several limitations of the present study should be acknowledged. First, while our institutional clinical cohort focused strictly on oral squamous cell carcinoma (OSCC), the public databases used for in silico analyses derived data from broader head and neck squamous cell carcinoma (HNSCC) populations, meaning anatomical subsite heterogeneity may introduce confounding. Second, baseline clinicopathological imbalances were observed (Table 1), as patients in the high-DAPK1 group were significantly younger and exhibited lower pN categories. To account for this, a pre-specified fully adjusted forced-entry model was designated as the primary multivariate analysis. Although the protective direction of the effect remained stable (HR = 0.51, *p* = 0.126), its statistical significance was attenuated due to the statistical power constraints of analyzing 31 death events across multiple covariates. This emphasizes that while DAPK1 holds prognostic potential, its complete independence from age and nodal status remains to be definitively established in larger, matched-cohort prospective validations. Third, a notable disconnect exists between our in vitro targeted-therapy model and the clinical cohort. Because clinical data regarding tumor responses to anti-EGFR agents (e.g., gefitinib or cetuximab) were unavailable in our surgical cohort, definitive therapeutic recommendations based solely on baseline DAPK1 expression cannot yet be formulated. Fourth, regarding experimental validation, the in vitro experiments were conducted using the FaDu hypopharyngeal carcinoma cell line rather than an OSCC-specific line (e.g., SAS or HSC-3), which limits the direct anatomical translation of these findings. Furthermore, different DAPK1 antibodies were utilized for Western blot and immunofluorescence analyses without formal cross-validation, representing a technical limitation. Additionally, our analyses utilized an antibody targeting total caspase-3 rather than its cleaved, active form; while this indicates an enrichment of the pro-apoptotic machinery, it does not directly confirm active apoptotic execution in situ. Fifth, because DAPK1 MFI was measured across whole fields without strict tumor-compartment restriction, the observed associations between DAPK1 and tumor-infiltrating lymphocytes may partly reflect intrinsic DAPK1 expression within the lymphocytes themselves. Additionally, no formal adjustment for multiple statistical testing was applied in exploratory subgroup comparisons, which warrants cautious interpretation.

## 5. Conclusions

In summary, the present findings support DAPK1 protein expression as a candidate prognostic biomarker associated with better overall survival, enrichment of the pro-apoptotic machinery, and increased T-cell infiltration in OSCC, while the mechanistic relationship between DAPK1, EGFR signaling, and immune regulation remains to be validated in future compartment-specific and prospective studies.

## Figures and Tables

**Table 1:** Demographic data.

Characteristics	All (n = 75)	DAPK1 High (n = 36)	DAPK1 Low (n = 39)	*p*-Value
	n (%)	n (%)	n (%)	
Age (Mean (SD))	56.68 (10.20)	54.11 (7.65)	59.05 (11.69)	0.033
Sex				
Female	10 (13.3)	5 (13.9)	5 (12.8)	1.000
Male	65 (86.7)	31 (86.1)	34 (87.2)	
Alcohol consumption				0.799
Never	20 (26.7)	9 (25.0)	11 (28.2)	
Ever	55 (73.3)	27 (75.0)	28 (71.8)	
Betel nut chewing				0.471
Never	27 (36.0)	11 (30.6)	16 (41.0)	
Ever	48 (64.0)	25 (69.4)	23 (59.0)	
Cigarette smoking				0.227
Never	13 (17.3)	4 (11.1)	9 (23.1)	
Ever	62 (82.7)	32 (88.9)	30 (76.9)	
Tumor subsite				0.76
Tongue	33 (44.0)	15 (41.7)	18 (46.2)	
Buccal mucosa	20 (26.7)	10 (27.8)	10 (25.6)	
Gingiva	14 (18.7)	7 (19.4)	7 (17.9)	
Floor of mouth (FOM)	4 (5.3)	2 (5.6)	2 (5.1)	
Retromolar trigone	2 (2.7)	2 (5.6)	0 (0.0)	
Hard palate	1 (1.3)	0 (0.0)	1 (2.6)	
Lower lip	1 (1.3)	0 (0.0)	1 (2.6)	
Differentiation				
Well	11 (14.7)	3 (8.3)	8 (20.5)	0.169
Moderately	60 (80.0)	32 (88.9)	28 (71.8)	
Poorly	4 (5.3)	1 (2.8)	3 (7.7)	
pT category				
T1	26 (34.7)	17 (47.2)	9 (23.1)	0.113
T2	14 (18.7)	7 (19.4)	7 (17.9)	
T3	5 (6.7)	2 (5.6)	3 (7.7)	
T4	30 (40.0)	10 (27.8)	20 (51.3)	
pN category				
N0	58 (77.3)	30 (83.3)	28 (71.8)	0.045
N1	8 (10.7)	5 (13.9)	3 (7.7)	
N2	9 (12.0)	1 (2.8)	8 (20.5)	
Adjuvant treatment				
Nil	40 (53.3)	22 (61.1)	18 (46.2)	0.078
Radiotherapy or chemotherapy	26 (34.7)	8 (22.2)	18 (46.2)	
Chemoradiotherapy	9 (12.0)	6 (16.7)	3 (7.7)	
Perineural invasion				
Absent	63 (84.0)	32 (88.9)	31 (79.5)	0.351
Present	12 (16.0)	4 (11.1)	8 (20.5)	
Lymphovascular invasion				
Absent	74 (98.7)	36 (100.0)	38 (97.4)	1.000
Present	1 (1.3)	0 (0.0)	1 (2.6)	
Extranodal extension				
Absent	67 (89.3)	32 (88.9)	35 (89.7)	1.000
Present	8 (10.7)	4 (11.1)	4 (10.3)	
Tumor thickness				
≤4 mm	25 (33.3)	16 (44.4)	9 (23.1)	0.085
>4 mm	50 (66.7)	20 (55.6)	30 (76.9)	
Surgical margin				
Negative	71 (94.7)	34 (94.4)	37 (94.9)	1.000
Positive	4 (5.3)	2 (5.6)	2 (5.1)	

**Table 2:** Univariate and multivariate analysis for overall survival.

Characteristics	n	Univariate Analysis		Secondary Multivariate Analysis (Stepwise)		Primary Multivariate Analysis (Forced-Entry)	
		HR (95% CI)	*p*-Value	HR (95% CI)	*p*-Value	HR (95% CI)	*p*-Value
Age		1.04 (1.00–1.07)	0.038			1.02 (0.99–1.05)	0.247
Sex							
Female	10	1					
Male	65	1.19 (0.42–3.41)	0.745				
Differentiation							
Well- Moderately	71	1					
Poorly	4	2.54 (0.77–8.40)	0.127				
pT category							
T1-T2	40	1		1			
T3-T4	35	3.62 (1.66–7.87)	0.001	2.68 (1.19–6.06)	0.018	2.50 (1.06–5.89)	0.036
pN category							
N0-N1	66	1					
N2	9	3.44 (1.48–8.01)	0.004			1.59 (0.62–4.07)	0.333
Adjuvant treatment							
Nil	40	1					
Radiotherapy or chemotherapy	26	2.35 (1.08–5.12)	0.032				
Chemoradiotherapy	9	2.28 (0.79–6.57)	0.127				
Perineural invasion							
Absent	63	1					
Present	12	3.16(1.45–6.89)	0.004				
Lymphovascular invasion							
Absent	74	1					
Present	1	9.92(1.22–80.63)	0.032	— *	— *		
Extranodal extension							
Absent	67	1					
Present	8	2.37 (0.91–6.19)	0.077				
Tumor thickness							
≤4 mm	25	1					
>4 mm	50	4.24 (1.48–12.13)	0.007				
Surgical margin							
Negative	71	1					
Positive	4	0.61 (0.08–4.48)	0.628				
DAPK1							
Low	39	1		1			
High	36	0.31 (0.14–0.69)	0.004	0.41 (0.18–0.91)	0.029	0.51 (0.21–1.21)	0.126

Abbreviations: HR, hazard ratio; CI, confidence interval; DAPK1, death-associated protein kinase 1. * Excluded from multivariate Cox regression analysis prior to model selection due to low event count (present in only 1 patient) and statistical instability, despite demonstrating a univariate *p*-value < 0.1.

**Table 3:** The association between DAPK1, CD4, CD8, and CASP3.

	DAPK1 Low	DAPK1 High	*p*-Value
CD4			
Low	23 (59.0)	7 (19.4)	0.001
High	16 (41.0)	29 (80.6)	
CD8			
Low	32 (82.1)	7 (19.4)	<0.001
High	7 (17.9)	29 (80.6)	
CASP3			
Low	29 (74.4)	5 (13.9)	<0.001
High	10 (25.6)	31 (86.1)	

Pearson chi-square analysis was used for discrete variables. Abbreviations: CASP3, Caspase 3. Percentages represent the proportion of patients within the respective DAPK1 expression group.

## Data Availability

The data that support the findings of this study are available upon request from the corresponding author. The data are not publicly available because of privacy or ethical restrictions.

## Supplementary Materials

The following supporting information can be downloaded at: https://www.mdpi.com/article/10.3390/cells15171566/s1. Figure S1: Flow chart showing the study’s enrollment; Figure S2: Differential expression of DAPK1 in various tumor types and corresponding normal tissues from TCGA database; Figure S3: Comparison of DAPK1 promoter methylation levels between normal tissues and primary tumors in head and neck squamous cell carcinoma (HNSCC) based on TCGA data; Table S1: Proportional hazard assumption evaluation; Table S2: Predictive performance and continuous risk associations of DAPK1, Caspase-3, CD4, and CD8.

## Abbreviations

CASP3	Caspase-3
DAPK1	Death-associated protein kinase 1
EGFR	Epidermal growth factor receptor
HNSCC	Head and neck squamous cell carcinoma
HPV	Human papillomavirus
IL-15	Interleukin-15
IMRT	Intensity-modulated radiotherapy
MFI	Mean fluorescence intensity
OS	Overall survival
OSCC	Oral squamous cell carcinoma
pN	Pathological nodal
pT	Pathological T
ROC	Receiver operating characteristic
STROBE	Strengthening the reporting of observational studies in epidemiology
TCGA	The Cancer Genome Atlas
TMA	Tissue microarray
