# Supplementary material for: Prognostic Relevance and Immune Correlates of DAPK1 Expression and CD4+/CD8+ T-Cell Infiltration in Oral Squamous Cell Carcinoma
Source: Cells. 2026 Aug 28;15(17):1566. doi: 10.3390/cells15171566 (PMC13565161; doi:10.3390/cells15171566)
Supplement: Supplementary file 1 [file cells-15-01566-s001.zip › Supplementary Table 2-V2.pdf]

Supplementary Table S2. Predictive performance and continuous risk associations of DAPK1, Caspase-3, CD4, and CD8.

Marker	Median Cut-off (MFI)	Optimal Cut-point (Youden)	AUC (95% CI)*	Continuous Cox HR (95% CI)**	p-value	Patients Reclassified by Youden Cut-point	Youden Cut-point HR (95% CI)***	Youden Cut-point p-value
DAPK1	5552	3459	0.63 (0.48 – 0.75)	0.64 (0.40 – 1.01)	0.054	14 (from Low to High)	0.41 (0.20 – 0.84)	0.015
Caspase-3	9265.5	13268.5	0.68 (0.55 – 0.80)	0.54 (0.32 – 0.92)	0.023			
CD4	14563.5	21398	0.63 (0.50 – 0.75)	0.85 (0.51 – 1.41)	0.528			
CD8	7558	7548.5	0.72 (0.60 – 0.83)	0.41 (0.23 – 0.74)	0.003			

* ROC analysis was performed using 5-year overall survival status, with survival as the positive classification. ** HR represents the hazard ratio per 1-SD increase in the marker MFI. *** For DAPK1, the Youden-derived optimal cut-point was 3459 MFI; the HR represents the comparison of patients with DAPK1 MFI ≥3459 versus <3459.
