# Supplementary material for: Prognostic Relevance and Immune Correlates of DAPK1 Expression and CD4+/CD8+ T-Cell Infiltration in Oral Squamous Cell Carcinoma
Source: Cells. 2026 Aug 28;15(17):1566. doi: 10.3390/cells15171566 (PMC13565161; doi:10.3390/cells15171566)
Supplement: Supplementary file 1 [file cells-15-01566-s001.zip › S_file_0807_V2.pdf]

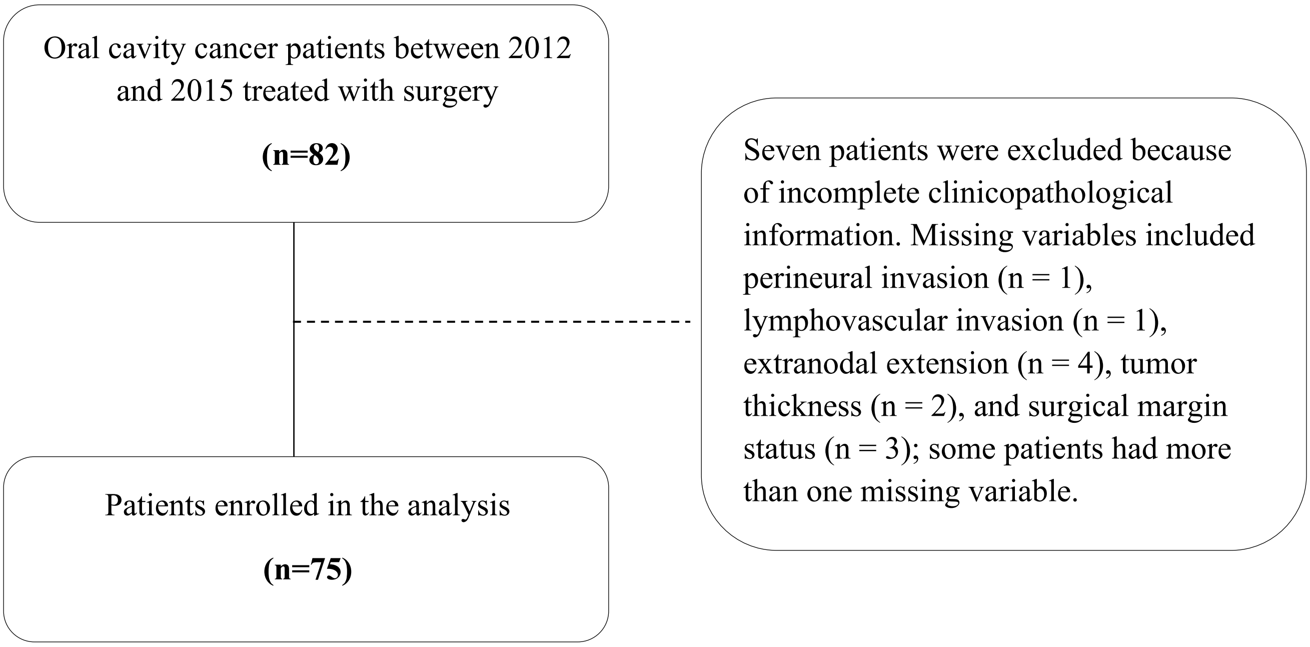

Supplementary Figure S1. Flow chart showing the study's enrollment.

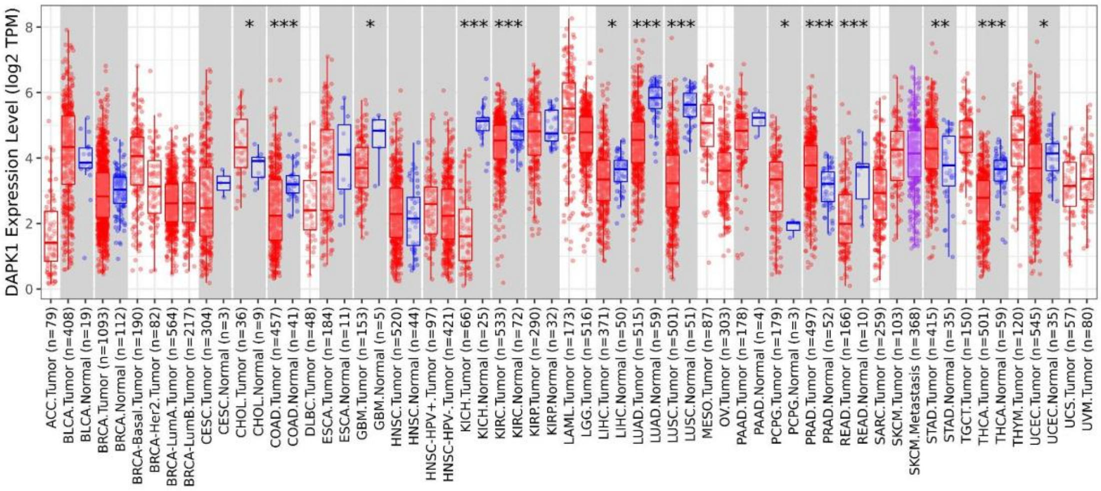

Supplementary Figure S2. Differential expression of DAPK1 in various tumor types and corresponding normal tissues from TCGA database.

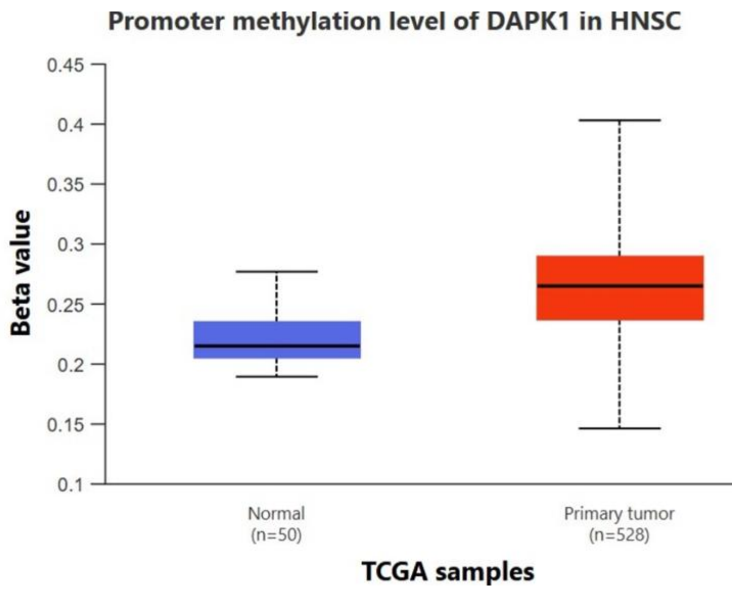

Supplementary Figure S3. Comparison of DAPK1 promoter methylation levels between normal tissues and primary tumors in head and neck squamous cell carcinoma (HNSCC) based on TCGA data.

Supplementary Table S1. Proportional hazard assumption evaluation.

Characteristics	PH assumption *p*-value
Age	0.687
Sex	
Female	0.959
Male	
Differentiation	
Well- Moderately	0.895
Poorly	
pT category	
T1-T2	0.321
T3-T4	
pN category	
N0-N1	0.301
N2	
Adjuvant treatment	
Nil	0.570
Radiotherapy or chemotherapy	
Chemoradiotherapy	
Perineural invasion	
Absent	0.455
Present	
Lymphovascular invasion	
Absent	0.379
Present	
Extranodal extension	
Absent	0.106
Present	
Tumor thickness	
≤4 mm	0.777
>4 mm	
Surgical margin	
Negative	0.437
Positive	
DAPK1	
Low	0.734
High	

Abbreviations: DAPK1, death-associated protein kinase 1.
